# Inadequate proximal screw fixation increases risk of failure following plate fixation of diaphyseal humerus fractures

**DOI:** 10.1186/s13018-023-03566-2

**Published:** 2023-02-27

**Authors:** Manisha R. Mistry, Jimmy Tat, Rafi Husain, Ujash Sheth, Robin R. Richards, Diane Nam

**Affiliations:** 1 Present Address: J159 - Division of Orthopaedic Surgery, The Ottawa Hospital Civic Campus, The University of Ottawa, Ottawa, ON K1Y 4E9 Canada; 2 Department of Surgery, Division of Orthopaedic Surgery, University of Toronto, Toronto, ON Canada; 3 Sunnybrook Orthopaedic Upper Limb (SOUL), Sunnybrook Health Sciences Centre, University of Toronto, Toronto, ON Canada

**Keywords:** Humerus shaft, Plate fixation, Fracture, Periprosthetic failure, Aseptic failure, Complications

## Abstract

**Background:**

Operative treatment of humeral shaft fractures (AO/OTA 12) is being performed more frequently. Accordingly, it is important to understand the complications associated with plate fixation. This study analyzes risk factors associated with mechanical failure following plate fixation of humeral shaft fractures in order to further elucidate the mode and location of failure.

**Methods:**

A retrospective review of 351 humeral shaft fractures was completed at a single level I trauma center. Eleven of eighty-five humeral shaft fractures had aseptic mechanical failure requiring revision (12.9%), following initial plate fixation. Fracture characteristics (AO type, comminution, location) and fracture fixation (plate type, multiplanar, number of screws proximal and distal to the fracture) were compared between aseptic mechanical failure and those without failure. A forward stepwise logistic regression analysis was performed to determine any significant predictors of aseptic mechanical failure.

**Results:**

There was significant differences in fixation between the aseptic mechanical failure group and those without failure, specifically in the number of screws for proximal fixation (*p* = 0.008) and distal fixation (*p* = 0.040). In the aseptic mechanical failure group, patients tended to have less than < 8 cortices of proximal fixation (82%) and less than < 8 cortices of distal fixation (64%). Conversely, in patients without mechanical failure there was a tendency to have greater than > 8 cortices in both the proximal (62%) and distal fixation (70%). A forward stepwise logistic regression analysis found that less than < 8 cortices of proximal fixation was a significant predictor of aseptic failure, OR 7.96 (*p* = 0.011). We think this can be accounted for due to the variable bone quality, thinner cortices and multiple torsional forces in the proximal shaft that may warrant special consideration for fixation.

**Conclusion:**

The current dogma of humeral shaft fracture stabilization is to use a minimum of 3 screws proximal and distal to the fracture, however the current study demonstrates this is associated with higher rates of mechanical failure. In contrast, 4 bicortical screws or more of fixation on either side of the fracture had lower failure rates and may help to reduce the risk of mechanical failure.

*Level of Evidence* Level III.

## Introduction

Diaphyseal humerus fractures (AO/OTA 12) have traditionally been managed non-operatively with splinting and functional bracing [1]. However, this treatment paradigm is being challenged, with increasing rates of operative fixation [2]. When operative fixation is employed, it is critical to understand appropriate fixation strategies to achieve bony union and decrease rates of failure.

Operative management can include plate fixation and intermedullary nailing. Plate fixation provides good results including a high rate of union, good functional scores, and low complication rates; in contrast to nail fixation which has been associated with higher shoulder dysfunction and reoperation rates and is indicated mostly for pathologic or highly comminuted fractures [3]. Common complications for open reduction internal fixation (ORIF) of humeral shaft fractures include radial nerve injury, infection, and hardware failure [4]. Recent studies have shown implant failure rates after plate fixation of humerus shaft fractures with a range from 2 to 6% [3–5]. When hardware failure occurs, it is important to understand how and where plate fixation fails and factors contributing to this failure. Ideal plate and screw fixation construct for open reduction and internal fixation of humeral shaft fractures remains controversial. Although 6–8 cortices of proximal and distal fixation have been recommended based on expert opinion [1], there is considerable variation in plating techniques and fixation constructs to match the variability in patient and fracture characteristics. Previous biomechanical work on optimal screw configurations, plate type and the use of locking constructs have been studied as a result, but they investigate a very narrow range of fracture patterns and configurations [6, 7]. The information garnered from these studies does not reflect the clinical scenarios routinely faced by surgeons.

This study aims to identify potential risk factors for mechanical failure following humeral shaft ORIF in order to further elucidate the mode and location of failure. We reviewed all patients who underwent humeral shaft ORIF over a 9-year period to evaluate modes and location of failure by fracture type and fixation construct, as well as to determine the risk factors contributing to failure.

## Methods

The study was approved by the institutional research ethics board (REB). We performed a retrospective electronic chart review of all patients who underwent operative plate fixation (ORIF) for diaphyseal humeral fractures from 2010 to 2019 at a single, level-one trauma center. Inclusion criteria included all AO/OTA humeral shaft fractures (AO/OTA 12) that were operatively treated with plate fixation (ORIF) in patients > 18 years of age. This included acute fractures and symptomatic non-union or malunion after failed non-operative treatment of humerus shaft fractures. Patients with pathological fractures, previous injury or surgery to the ipsilateral humerus, use of bone graft, nerve or vascular injury aside from radial nerve palsy, or patterns with extension into the proximal or distal segments of the humerus were excluded. Additionally, patients who did not have adequate follow-up to bony union and clinical discharge were also excluded, as failure prior to union could not be captured.

Charts were reviewed by three independent reviewers. Clinical notes and radiographs were reviewed from the time of initial trauma to our predetermined end point for analysis. This was defined as either (a) mechanical failure requiring revision fixation, or (b) successful bony and radiographic union accompanied by clinical discharge by the treating surgeon. The following characteristics were described and quantified for each patient, under the main categories of patient, fracture and fixation characteristics. Patient characteristics included demographic information and presence of associated soft tissue injury (i.e., open fracture status and grade), additional injuries (i.e., polytrauma), and presence of severe polytrauma defined by an Injury Severity Score (ISS) > 15.

Initial injury radiographs were used to obtain fracture characteristics, including classification, presence of comminution, location and extent of the fracture. Fractures were classified by AO/OTA subtype and identify the presence of comminution defined as 3 or more fragments. We defined the humeral diaphysis as the length between the surgical neck of the humerus to the supracondylar ridge. Using this measurement, we were able to define the percent of shaft involved in the fracture by dividing the measured length of the fracture by the length of the shaft. Based on the total length of the shaft, we could define the proximal (0–33%), middle (33–66%) and distal (66–100%) thirds of the diaphysis. Fractures were classified as proximal, middle or distal third fractures if the majority of the length of the fracture was located within that division of the diaphysis. Measurements were averaged between all three reviewers.

Finally, radiographs and operative reports were reviewed to confirm fixation characteristics. These included plate type based on size of screw and plate design (3.5-mm vs. 4.5-mm, broad vs narrow plate). Plate length was determined by the number of available holes for fixation. Fixation strategy was specified including bridging vs rigid fixation (compression plating), use of multiplanar or locking fixation, and cortices engaged above and below the fracture site. A minimum of 8 cortices of fixation was set as a threshold value for adequate fixation as it represents at least 4 bicortical screws, and allowed us to look at a possible difference between 3 and 4 bicortical screws on either side of the fracture. Surgical approaches varied depending on fracture location. Fractures in the proximal 1/3 used the deltopectoral approach, middle 1/3 and distal 1/3 fractures used either a triceps split, paratricipital, or posterolateral approach.

End points were defined as mechanical failure requiring revision fixation or discharge from follow up secondary to radiographic and clinical healing of the fracture. Mechanical failure included screw pull-out, plate bending or breakage and loss of alignment of fracture requiring revision surgery. Failures were independently assessed for cause of failure which was qualitatively described noting presence and location of screw pullout, screw or plate breakage and suboptimal initial alignment or fixation strategies based on basic fracture fixation principles.

### Statistical analysis

Qualitative variables were expressed as frequencies and percentages. Fisher’s Exact Test was used to compare categorical data including the rates of polytrauma, presence of open fracture, fracture failure, fracture characteristics such as AO/OTA classification, fixation construction, presence of comminution, and fracture % involvement of humeral shaft by quartile, fracture location, and fixation above and below the fracture. For fracture fixation, both above and below the fracture, we reported whether the fracture fixation included at least 8 screws of fixation. Mean values of age were compared utilizing independent-sample t-tests. A forward stepwise logistic regression analysis was performed to determine which significant variables from the univariate analysis could be modeled as predictors of aseptic failure. Statistical significance was set at *p* < 0.05. Data were analyzed using Stata/IC 16.1 (StataCorp TX, USA).

## Results

A total of 351 patients were initially screened and 85 patients met inclusion criteria, including 45 females and 40 males with a mean age of 47 years (IQR 33). The majority (73%) of patients who underwent operative fixation had multiple injuries. Severely polytraumatized patients (ISS > 15) represented approximately one third (35%) of all eligible patients. These factors were not found to significantly influence rates of aseptic mechanical failure (*p* > 0.05). We identified 13 patients (15%) who required revision surgery, 2 for infection (2.4%), and 11 for aseptic mechanical failure (13%). The mean time from index procedure to revision surgery was 174 days. There were no differences between groups in rates of aseptic revision by age (*p* = 0.89) or sex (*p* = 0.067). A significant increase in the rate of aseptic revision was found for patients with open fractures (*p* = 0.045) (Table 1).

**Table 1:** Demographic characteristics of patients requiring revision for aseptic mechanical failure (Group 1), and those who did not (Group 2)

	n (%)	Group 1	Group 2	*p* Value
N = 85		11	74	
Sex				
Male		6 (15)	34 (85)	*p* = 0.749
Female		5 (11)	40 (89)	
Mean age (IQR)	47 (33)	53 (35)	46 (44)	*p* = 0.890
Trauma characteristics				
Open fracture	12 (14)	4 (36)	8 (67)	*p* = 0.045*
Polytrauma	62 (73)	9 (82)	53 (72)	*p* = 0.719
ISS > 15	30 (35)	6 (55)	24 (32)	*p* = 0.184

P value derived from Student’s T test for continuous variables (age) and Fischer’s Exact Test for remaining categorical variables, *p* < 0.05*

There were significant differences in fixation characteristics between the aseptic mechanical failure group and those without failure, specifically in the number of screws for proximal fixation (*p* = 0.008) and distal fixation (*p* = 0.040) (Table 2). In the aseptic mechanical failure group, patients tended to have less than < 8 cortices of proximal fixation (82%) and less than < 8 cortices of distal fixation (64%). Conversely, in patients without mechanical failure there was a tendency to have greater than > 8 cortices in both the proximal (62%) and distal fixation (70%). Otherwise, the remainder of fracture characteristics and implant types were not significant between groups (*p* > 0.05). This included humerus shaft fracture AO/OTA classification, fracture comminution, fixation type (bridge vs rigid), fracture location, percentage of shaft involvement, implant type, and multi-planar fixation (Table 2). Tables 3 and 4 show the effect of inadequate proximal and distal fixation according to location of fracture, with middle third (*p* = 0.045) and distal third (*p* = 0.031) diaphyseal fractures having a significantly higher rate of mechanical failure when fixed with less than 8 cortices of proximal fixation (Table 3). Additionally, distal third diaphyseal fractures also had a higher failure rate when fixed with less than 8 cortices of fixation distally (*p* = 0.046, Table 4).

**Table 2:** Radiographic and fixation characteristics in patients requiring revision for aseptic mechanical failure (Group 1), and those who did not (Group 2)

	Group 1	Group 2	*p* Value
N = 85	11	74	
AO/OTA classification			
n (%)			
12A	6 (55)	38 (51)	0.795
12B	5 (45)	28 (38)	
12C	0 (0)	8 (11)	
Comminution			
Yes	6 (55)	45 (61)	0.748
No	5 (45)	29 (39)	
Fixation type			
Rigid	10 (91)	68 (92)	1.000
Bridge	1 (9)	6 (8)	
Fracture location			
Proximal 1/3	1 (9)	5 (7)	0.767
Middle 1/3	7 (64)	51 (69)	
Distal 1/3	3 (27)	18 (24)	
% Involvement of shaft			0.068
0–25%	11 (100)	49 (67)	
26–50%	0 (0)	22 (30)	
51–75%	0 (0)	2 (3)	
76–100%	0 (0)	0 (0)	
Implant type			0.194
3.5 mm LCDC plate	4 (36)	9 (12)	
4.5 mm narrow LCDC plate	1 (9)	7 (9)	
4.5 mm broad LCDC plate	0 (0)	15 (20)	
Proximal humeral locking plate	3 (27)	19 (26)	
Posterolateral distal humeral Plate	3 (27)	24 (32)	
Muti-planar fixation			0.329
Yes	9 (82)	67 (91)	
No	2 (18)	7 (9)	
≥ 8 Cortices proximal fixation			0.008*
No	9 (82)	28 (38)	
Yes	2 (18)	46 (62)	
≥ 8 Cortices distal fixation			0.040*
No	7 (64)	22 (30)	
Yes	4 (36)	52 (70)	

Bold values are statistically significant

*p* Value derived from Fisher’s Exact Test, *p* < 0.05 *

Radiographic and fixation characteristics in patients requiring revision for aseptic mechanical failure (Group 1), and those who did not (Group 2) using Fisher’s Exact Test. The table demonstrates that factors that were significantly different between the two groups were proximal and distal fixation. We show that the mechanical failure group tended to have less than < 8 cortices of proximal fixation (*p* = 0.008) and less than < 8 cortices of distal fixation (*p* = 0.04)

**Table 3:** Rate of aseptic mechanical failure vs no failure by ≥ 8 cortices of proximal fixation and fracture location

Fracture location	Group	≥ 8 Cortices proximal fixation		*p* Value
		Yes (%)	No (%)	
Proximal 1/3	Mechanical failure No mechanical failure	1 (100) 4 (80)	0 (0) 1 (20)	1.0
Middle 1/3	Mechanical failure No mechanical failure	1 (14) 21 (42)	6 (86) 29 (58)	0.045*
Distal 1/3	Mechanical failure No mechanical failure	0 (0) 13 (77)	3 (100) 4 (23)	0.031*

Bold values are statistically significant

*p* Value derived from Fisher’s Exact Test, *p* < 0.05*

Comparison of rate of aseptic mechanical failure vs no failure according to proximal fixation (≥ 8 cortices) and fracture location using a Fisher’s Exact Test. The table shows higher rates of mechanical failure when proximal fixation had less than < 8 cortices; specifically, in fracture locations at the middle 1/3 and distal 1/3 humerus shaft fractures. Mechanical failure in proximal 1/3 fractures did not appear to be affected by number of screws in the proximal fixation. We think this may have been confounded by the type of construct used in proximal fractures (typically proximal humerus internal locking plates and locking screws)

**Table 4:** Rate of aseptic mechanical failure vs no failure with by ≥ 8 cortices of distal fixation and fracture location

Fracture location	Group	≥ 8 Cortices proximal fixation		*p* value
		Yes (%)	No (%)	
Proximal 1/3	Mechanical failure No mechanical failure	0 (0) 2 (40)	1 (100) 3 (60)	1.0
Middle 1/3	Mechanical failure No mechanical failure	3 (43) 34 (68)	4 (57) 16 (32)	0.226
Distal 1/3	Mechanical failure No mechanical failure	1 (33) 16 (94)	2 (67) 1 (6)	0.046*

Bold value is statistically significant

*p* Value derived from Fisher’s Exact Test, *p* < 0.05*

Comparison of rate of aseptic mechanical failure versus no failure distal fixation (≥ 8 cortices) and fracture location using Fisher’s Exact Test. The table shows higher rates of mechanical failure when distal fixation had less than < 8 cortices; specifically, when the fracture was in the distal 1/3 humerus (*p* = 0.046). Whereas, fractures that were located in the proximal 1/3 and middle 1/3 of the humerus did not appear to be affected by number of screws in the distal fixation (*p* > 0.05)

In order to determine which factors could be modeled as predictors of aseptic mechanical failure, a forward stepwise logistic regression analysis was performed using the two variables found to be significantly different between groups from our univariate analysis: (1) the presence of 8 or more cortices of proximal fixation and (2) the presence of 8 or more cortices of distal fixation. The forward stepwise regression demonstrated that inadequate proximal fixation was significantly associated with eightfold higher odds of aseptic failure (OR 7.96, confidence interval [CI] 1.59–39.7, *p* = 0.011). A minimum of 8 cortices of distal fixation was not found to be a significant predictor and was therefore removed from the model.

## Discussion

With increasing rates of operative fixation of diaphyseal humeral fractures [2, 4], it is important to understand the ideal techniques to avoid failure and complications in patients undergoing operative fixation. To our knowledge, this is the first study to investigate risk factors for aseptic mechanical failure in humerus shaft plate fixation. We sought to develop a greater understanding of how different fracture characteristics may demand a deviation from historic guidelines, and how to technically optimize fixation. We observed an overall revision rate for mechanical failure of 13%. Our findings are consistent with previous reports which have observed revision rates ranging from 2 to 30% [8, 9].

The current study demonstrated a significant rate of aseptic mechanical failure in constructs with less than 8 cortices of proximal or distal fixation (*p* < 0.05). Previous reports have suggested a minimum of 6–8 cortices of fixation on either side of a humeral shaft fracture undergoing plate fixation [1, 10, 15]. Our results suggest that fixation constructs with 4 bicortical screws or more of fixation on either side of the fracture had lower failure rates than those fixed with 3 screws a side. Although we cannot comment on any potential advantage of having more than 4 screws a side, surgeons are routinely using more fixation, tailored to the specific fracture type, which can often include longer constructs or multiple plates. To our knowledge, there are no studies that directly compare 6 to 8 cortices of fixation in diaphyseal humerus fracture.

**Fig. 1:**
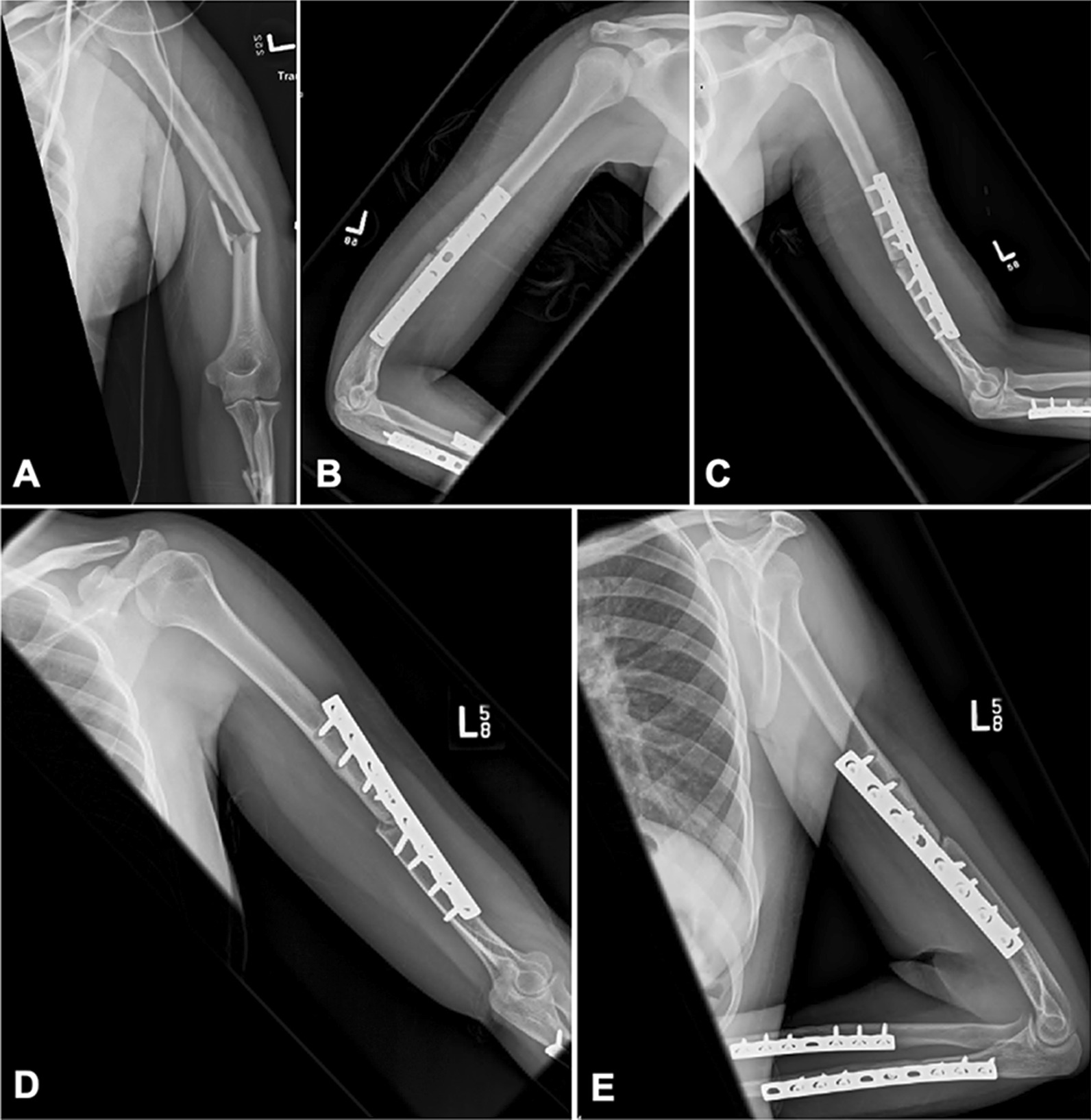
25-year-old female with a middle 1/3 diaphyseal, AO/OTA 12B2 humerus fracture (**A**). Patient received ORIF with Synthes narrow 4.5 mm LCDC plate, with immediate post-operative radiographs shown (**B**, **C**). Radiographs demonstrate unicortical fixation of one of the proximal screws, and 5 cortices of proximal fixation (**C**). Two years late the patient went on to mechanical failure with proximal screw pullout (**D**, **E**), requiring revision

When comparing proximal and distal fixation, fixation of the proximal segment may be more critical in minimizing mechanical failure. We found inadequate proximal fixation, in the form of less than 8 cortices of fixation, to be a significant predictor of failure (*p* = 0.011), with an eight-fold increase in the rate of mechanical failure compared to constructs with 8 or more cortices of proximal fixation (OR 7.96). Secondary analysis of failure by fracture location (proximal, middle and distal third), was also conducted. Significantly higher rates of failure were seen in patients with middle (*p* = 0.45) and distal third fractures (*p* = 0.31) with less than 8 cortices of proximal fixation. Additionally, we observed that among those with middle third shaft fractures, failure occurred frequently in the form of proximal screw pull out (Fig. 1D, E). Table 5 demonstrates the mode of failure according to fracture location and fixation type. We hypothesize these findings are due to features unique to the proximal humerus. Firstly, the proximal humeral diaphysis is directly enacted on by multiple deforming forces including coronal and sagittal plane forces. In addition, it is uniquely subjected to rotational torque forces applied by the teres major and minor, infraspinatus, subscapularis, pectoralis major and latissimus dorsi [16]. Adequate fixation must overcome and withstand these multiplanar forces on the proximal segment. Secondly, it has been well documented that the proximal humeral cortical diaphysis thickness is variable and has decreased cortical thickness in osteoporotic bone [17]. As a result, special attention must be paid to the type and amount of fixation in that segment to avoid proximal screw pull out.

**Table 5:** Fixation characteristics in patients requiring revision for aseptic mechanical failure

Subject	Fracture location	Plate	Number of screws		
			Proximal	Distal	Method of failure
1	Proximal 1/3	3.5 mm Proximal Humerus Locking	6*	4	Distal screw pull out
2	Middle 1/3	4.5 mm LCDC, narrow	3	3	Plate failure
3	Middle 1/3	3.5 mm LCDC, narrow	3	3	Plate failure
4	Middle 1/3	3.5 mm LCDC, broad	3	3	Proximal screw pull out
5	Middle 1/3	4.5 mm LCDC, broad	3	4	Plate failure
6	Middle 1/3	4.5 mm LCDC, broad	3	4	Proximal screw pull out
7	Middle 1/3	4.5 mm LCDC, broad	4	4	Proximal screw pull out
8	Middle 1/3	3.5 mm LCDC, narrow	3	3	Distal screw pull out
9	Distal 1/3	3.5 mm LCDC, narrow	3	4	
10	Distal 1/3	4.5 mm LCDC, narrow	3	3	Proximal screw pull out
11	Distal 1/3	3.5 mm LCDC, narrow + 1/3 tubular	3	3	Distal fragment failure

^*^Represents use of locking screws, LCDC = limited contact dynamic compression

Fixation characteristics in patients requiring revision for aseptic mechanical failure. The characteristics include location of fracture in the humeral shaft (proximal, middle, distal 1/3 of humerus) and the type of construct (plate fixation, and number of screws used in the proximal vs distal segment)

Regarding proximal third diaphyseal fractures, we failed to see a significant difference between those with 8 or more cortices of proximal fixation vs those with less than 8 cortices (*p* = 1.0). We attribute these equivocal findings to the preference of proximal humerus locking construct for fixation of these fractures, in which unicortical locking screws provide a mechanical advantage in a shorter proximal segment and allow for higher screw density. These constructs cannot be directly compared to non-locking plates used in more distal fractures. A higher rate of mechanical failure was also seen in distal third diaphyseal fractures with less than 8 cortices of distal fixation (*p* = 0.046). As distal fixation was not found to be a significant predictor of failure after regression analysis, the significance of this finding is indeterminant and we cannot draw conclusions regarding the number of cortices of distal fixation. Thus, 8 cortices of proximal fixation may be recommended, especially in middle and distal third diaphyseal fractures fixed with straight plates without proximal locking extension. It is important to ensure screws are truly bicortical if intended, as demonstrated by the patient in Fig. 1C, who went on to aseptic mechanical failure, with post-operative radiographs demonstrating that the screws were not fully engaged in the distal cortex.

There are several limitations to our study. The limited sample size may have resulted in a type II error. Although a large volume of patients underwent operative management of a diaphyseal humerus fracture during the study period, a significant proportion did not meet inclusion criteria. The principal reason for exclusion in the majority of cases was lack of follow-up to radiographic and clinical union. As a referral center for high volume trauma, many patients were either lost to follow-up, or transferred back to the referring centers for long-term follow-up after acute management of their injuries. As a result, we were also unable to account for patients who may have experienced fixation failure and presented to another institution for care. The remainder of patients who were eligible were followed until radiographic union and discharged from follow-up. We felt that this was adequate and did not pursue follow-up to one year, as our endpoint was defined as mechanical failure or discharge. The retrospective nature of the current study is also a limitation as it is subject to the inherent biases associated with this study design. Finally, we were unable to locate a comprehensive list of patient comorbidities as often these were not documented. Despite this, we did not find any significant demographic differences between the two groups, thereby improving the generalizability of our results.

## Conclusion

Our results suggest, diaphyseal humerus fractures (AO/OTA 12) treated with ORIF may benefit from fixation with 8 or more cortices of fixation proximally and distally to avoid mechanical failure. Special attention should be paid to proximal fixation in middle and distal third humeral shaft fractures to mitigate proximal pull out and mechanical failure due to inadequate fixation. Further biomechanical and prospective comparative studies are required to confirm failure patterns and determine optimal fixation constructs for diaphyseal fractures of the humerus.

## Data Availability

Not applicable.
